# Effects of CaCl_2_ Treatment Alleviates Chilling Injury of Loquat Fruit (*Eribotrya japonica*) by Modulating ROS Homeostasis

**DOI:** 10.3390/foods10071662

**Published:** 2021-07-19

**Authors:** Yuanyuan Hou, Ziying Li, Yonghua Zheng, Peng Jin

**Affiliations:** College of Food Science and Technology, Nanjing Agricultural University, Nanjing 210095, China; 2019208027@njau.edu.cn (Y.H.); 2017108025@njau.edu.cn (Z.L.); zhengyh@njau.edu.cn (Y.Z.)

**Keywords:** loquat fruit, calcium chloride, chilling injury, reactive oxygen species, ascorbate–glutathione cycle

## Abstract

The effects of calcium chloride (CaCl_2_) treatment on chilling injury (CI), reactive oxygen species (ROS) metabolism, and ascorbate-glutathione (AsA-GSH) cycle in loquat fruit at 1 °C storage for 35 d were investigated. The results indicated that CaCl_2_ treatment remarkably suppressed the increase in browning index and firmness as well as the decrease in extractable juice rate. CaCl_2_ treatment also decreased the production of superoxide radical ($O_{2}^{•-}$), hydrogen peroxide (H_2_O_2_) content, but increased the 1,1-diphenyl-2-picrylhydrazyl (DPPH), hydroxyl radical (${\mathrm{OH}}^{•}$) scavenging ability, the activities of superoxide dismutase (SOD), catalase (CAT), and their gene expressions. Moreover, compared to the control loquat fruit, CaCl_2_-treated fruit maintained higher contents of AsA, GSH, higher levels of activities of ascorbate peroxidase (APX), glutathione reductase (GR), dehydroascorbate reductase (DHAR), and monodehydroascorbate reductase (MDHAR) and expressions of *EjAPX*, *EjGR*, *EjMDHAR*, and *EjDHAR*, but exhibited lower glutathione disulfide (GSSG) content. These results suggested that CaCl_2_ treatment alleviated CI in loquat fruit through enhancing antioxidant enzymes activities and AsA-GSH cycle system to quench ROS.

## 1. Introduction

Loquat (*Eriobotrya japonica* Lindl.), as a type of non-climacteric fruit, presents a short postharvest life at ambient temperature because of its physiological deterioration and microbial decay. Refrigeration is widely adopted to retain quality and prolong the shelf life of loquat fruit. Nevertheless, loquat fruit is vulnerable to chilling injury (CI), displaying symptoms including enhanced fruit firmness, internal browning, and reduced extractable juice rate after long-term low temperature storage [1,2], which severely influences the texture and commercial value of the fruit, and eventually reduces consumer acceptance. Therefore, the exploration into the mechanism of CI in loquat fruit is of great significance.

It is well-established that the occurrence of chilling injury in higher plants is closely associated with oxidative stress resulted from excessive reactive oxygen species (ROS) such as superoxide anion ($O_{2}^{•-}$), hydrogen peroxide (H_2_O_2_), and hydroxyl radical (${\mathrm{OH}}^{•}$) [3]. This large quantity of ROS accumulation alter the membrane organization and cause or exacerbate lipid peroxidation, leading to damage to the cell system [4]. To protect against oxidative damage and maintain homeostasis of ROS, plants have evolved a complicated antioxidant system including enzymatic and non-enzymatic antioxidant components. Superoxide dismutase (SOD), catalase (CAT) and peroxidase (POD) are primary antioxidant enzymes, while ascorbate (AsA) and glutathione (GSH) are non-enzymatic antioxidants [5]. The ascorbate–glutathione (AsA-GSH) cycle, which mainly consists of ascorbate peroxidase (APX), glutathione reductase (GR), dehydroascorbate reductase (DHAR), and monodehydroascorbate reductase (MDHAR), plays a critical role in ROS elimination and preventing oxidative damage [6]. It has been reported that increasing the activities of antioxidant enzymes (SOD, CAT, APX, GR, MDHAR, and DHAR) to eliminate excessive ROS is beneficial for alleviating the occurrence of CI in many fruit, such as peach [7], litchi [8], and bell pepper [9]. Furthermore, previous studies have confirmed that the increased antioxidant enzymes activities (SOD, CAT, and POD) and AsA-GSH cycle and mitigated peroxidation of membrane lipids might contribute to the enhancement of chilling tolerance in loquat fruit [10,11]; nevertheless, their regulation mechanism at a molecular level remains unknown.

Calcium ion (Ca^2+^), as an essential nutrient for fruit, plays a crucial role in building the structure of cell wall and cellular membranes [12]. Moreover, Ca^2+^ is also a crucial second messenger in plant signal transduction involving in physiological processes and the responses to various stresses [13]. Under cold stress, induced transient elevations of cytosolic Ca^2+^ are sensed by different calcium binding proteins, consequently initiating various physiological responses in the cell [14]. Dong et al. [15] reported that overexpression of the *MdCPK1a* gene increased tobacco chilling tolerance by inducing the expression of *SOD*, *CAT*, *APX* and scavenging ROS accumulation. Li et al. [16] suggested that postharvest Ca^2+^ application significantly increased Ca^2+^ and calmodulin content, and concomitantly, endogenous GABA content in ‘Nanguo’ pear fruit, which in turn delayed fruit browning after low-temperature storage. In addition, recent studies have reported that calcium chloride (CaCl_2_) application can trigger antioxidant system activity and maintain ROS homeostasis to increase cold tolerance of postharvest vegetables and fruits. For instance, CaCl_2_ application effectively alleviated peel browning caused by chilling injury of pear fruit on account of the inhibition of membrane lipid peroxidation and a higher activity and expression of SOD [17]. Wei and Zhao [18] also found that CaCl_2_ treatment could alleviate chilling injury symptoms in winter jujube fruit by promoting the SOD, CAT, and POD activities to scavenge ROS. In green peppers, CaCl_2_ treatment significantly suppressed ROS levels by regulating activities of SOD, POD, CAT as well as promoting the AsA-GSH cycle and, in turn, enhanced chilling tolerance [19]. For loquat fruit, a previous study demonstrated that CaCl_2_ treatment could significantly enhance the cold tolerance of loquat fruit through regulating energy metabolism and accumulating osmotic substances [20]. However, whether the inhibitory impact of calcium application is associated with the modulation of the ROS-scavenging system for the chilling injury of loquat fruit remains unclear.

Therefore, the impact of exogenous CaCl_2_ treatment on the ROS metabolism and AsA–GSH cycle system in loquat fruit were examined, which aimed to elucidate the antioxidant system triggered by Ca^2+^ treatment in alleviating oxidative damage during CI. The results will extend the mechanistic understanding of CaCl_2_-inhibited chilling injury in postharvest loquat fruit, and supply a scientific basis for the application of CaCl_2_ treatment as a useful technology to prolong the storage life of vegetables and fruits.

## 2. Materials and Methods

### 2.1. Materials and Treatments

Loquat fruit (*Eriobotrya japonica* L. cv. ‘Changhong’) of uniform maturity, size, and color were harvested from a commercial orchard in Fujian, China. Fruit with no mechanical damage or disease were chosen and then randomly divided into two groups (375 fruits in each group). One percent of CaCl_2_ solution was employed to soak loquat fruit for 10 min as CaCl_2_ treatment group, with the concentration chosen according to Li et al. [20]. Distilled water was used to immerse fruits for 10 min as control group. After treatment, all fruit were stored at 1 ± 1 °C with 90−95% relative humidity for 35 days. A total of 75 fruits from each group comprising three replicates were randomly sampled at 7 days intervals during storage. Fruit firmness was evaluated using 10 fruits, and 35 fruits were cut into small pieces, frozen using liquid nitrogen and stored at −80 °C for subsequent analysis. At each sampling day, another 30 fruits were transferred from 1 °C and kept at 20 °C for 3 days to stimulate shelf condition, and then internal browning index, firmness and extractable juice were measured. Three independent replicates were evaluated.

### 2.2. Measurement of Browning Index, Firmness, and Extractable Juice

The browning index was visually assayed using the reported method [20]. Browning index was scored based on a 5-grade scale (0, no visible symptoms; 1, <5% of browning; 2, 5–25% of browning; 3, 25–50% of browning; 4, >50% of browning). The result was acquired by the equation given below:

(1) $$\mathrm{Browning}\;\mathrm{index}=\frac{\sum \left(\mathrm{scale}\;\mathrm{of}\;\mathrm{browning}\right)\times \left(\mathrm{number}\;\mathrm{of}\;\mathrm{fruit}\;\mathrm{at}\;\mathrm{the}\;\mathrm{scale}\right)}{\left(4\times \mathrm{total}\;\mathrm{number}\;\mathrm{of}\;\mathrm{fruit}\;\mathrm{in}\;\mathrm{each}\;\mathrm{tratment}\right)}\times 10$$

Fruit firmness was determined following the method of Zhang et al. [21]. Firmness from 10 loquat fruits selected randomly were assayed by a TA-XT2i texture analyzer (Stable Micro System Ltd., Surrey, UK), with a 5 mm diameter probe (test speed 1 mm s^−1^). Firmness measurement was operated at two opposite sides of fruit, and the result was expressed as N. 

Extractable juice was evaluated based on the description of Cao et al. [10]. The unit of % was used to express extractable juice rate.

### 2.3. Measurement of $O_{2}^{•-}$, Generation Rate and H_2_O_2_ Content

The generation rate of $O_{2}^{•-}$ and H_2_O_2_ content were assayed based on the description of Cao et al. [10]. The $O_{2}^{•-}$ generation rate was acquired based on standard curve obtained from sodium nitrite and denoted as the unit of nmol g^−1^ min^−1^ on the basis of fresh weight (FW). The H_2_O_2_ content was acquired based on a standard curve and represented as μmol g^−1^ FW. 

### 2.4. Measurement of 1,1-Diphenyl-2-Picrylhydrazyl (DPPH) and $OH^{•}$ Radical Scavenging Capacity

Frozen tissue (2 g) was employed to determine DPPH and ${\mathrm{OH}}^{•}$ radical scavenging capacity according to Wang et al. [22]. 

For DPPH radical scavenging rate, the reaction mixture contained 0.1 mL of crude enzyme and 1.9 mL of 120 μmol L^−1^ DPPH. The result was acquired using the equation given below:

(2) $$\mathrm{DPPH}\;\mathrm{radical}\;\mathrm{scavening}\;\mathrm{rate}\;\left(\%\right)=[\left(A_{0}-A_{1})/{\;A}_{0}\right]\times 100$$

A_0_ and A_1_ indicate the absorbance of control and sample, respectively. Percentage was used for expressing DPPH radical scavenging rate.

For ${\mathrm{OH}}^{•}$ scavenging rate, the reaction mixture included 0.5 mL of crude enzyme, 1.5 mL of salicylic acid, 2 mL of water and 0.1 mL of 0.3% H_2_O_2_. Results were calculated by the following equation:

(3) $$\mathrm{Hydroxyl}\;\mathrm{radical}\;\mathrm{scavening}\;\left(\%\right)=[\left(A_{0}-A_{1})/{\;A}_{0}\right]\times 100$$

A_0_ and A_1_ refer to absorbance of the control and samples, respectively. The unit of % was regarded as ${\mathrm{OH}}^{•}$ radical scavenging ability.

### 2.5. Determination of SOD and CAT Activities

Determination of SOD and CAT enzymes activities were carried out based on the description of Zhang et al. [21]. One unit of SOD activity was defined as the amount of enzyme causing 50% inhibition of nitroblue tetrazolium (NBT) reduction. One unit of CAT was defined as the amount of enzyme that decomposed 1 μmol of H_2_O_2_ min^−1^. The unit of U g^−1^ was used to express SOD and CAT activities on the basis of fresh weight. 

### 2.6. Measurement of Parameters Related to the AsA-GSH Cycle

Determination of AsA content was performed based on the description of Wang et al. [22], and the unit of AsA content was represented as mg g^−1^ FW based on the standard curve.

GSH and glutathione disulfide (GSSG) contents were assayed using assay kits (Solarbio, Beijing, China), according to manufacturer’s instructions. GSH and GSSG contents were represented as μg g^−1^ on the basis of fresh weight.

APX activity was performed following the procedures reported by Liu et al. [8]. The APX activity was acquired from the alteration in absorbance (290 nm) for 5 min promoted by the AsA after adding H_2_O_2_. One unit of APX activity was defined as the amount of enzyme that led to a 0.01 variation in absorbance every minute at 290 nm, and the APX activity was represented as U g^−1^ FW.

The activity of GR was determined through monitoring the variation in absorbance at 340 nm for 2 min due to oxidation of NADPH with GSSG [8], and the results were represented as U g^−1^ FW.

Frozen tissue (2 g) was used to measure DHAR and MDHAR activities according to the description of Cao et al. [10]. DHAR activity was acquired from the variation in absorbance at 265 nm and the result was represented as U g^−1^ FW. MDHAR activity was acquired through evaluating the reduction in NADH absorbance at 340 nm and represented as U g^−1^ FW.

### 2.7. Real-Time Quantitative PCR (RT-qPCR) Analysis

Total RNA was extracted from loquat fruit according to an improved CTAB method [23]. First-strand cDNA was synthesized using Hifair Ⅲ First Strand cDNA Synthesis Super Mix for qPCR (gDNA digester plus) (11141ES60, YEASEN, Shanghai, China). RT-qPCR analysis was conducted with a 7500 Fast Real-Time PCR System (Thermo Fisher Scientific, Waltham, MA, USA) by mixing the primers, Hieff qPCR SYBR Green Master Mix (11202ES08, YEASEN), cDNA template and RNase-free water in a total volume of 20 μL. *EjACT* was selected as the internal control to normalize the relative gene expression in each sample [24]. The relative expressions were normalized against the threshold cycle (Ct) value of the target gene and calculated with the 2^–ΔΔCt^ method. All analyses were conducted in triplicate, and all primer sequences were shown in Supplementary Table S1.

### 2.8. Statistical Analysis

Experiments were conducted at least three biological replicates and all data were expressed as mean ± standard error (SE). The statistical analyses were performed using IBM SPSS Statistics 22 (SPSS Inc., Chicago, IL, USA). The independent samples T-test was used to compare the data of control and CaCl_2_ treatment groups. The value of *p* < 0.05 indicated significant difference.

## 3. Results

### 3.1. Effects of CaCl_2_ Treatment on Internal Browning Index, Fruit Firmness, Extractable Juice of Loquat Fruit

**Figure 1:**
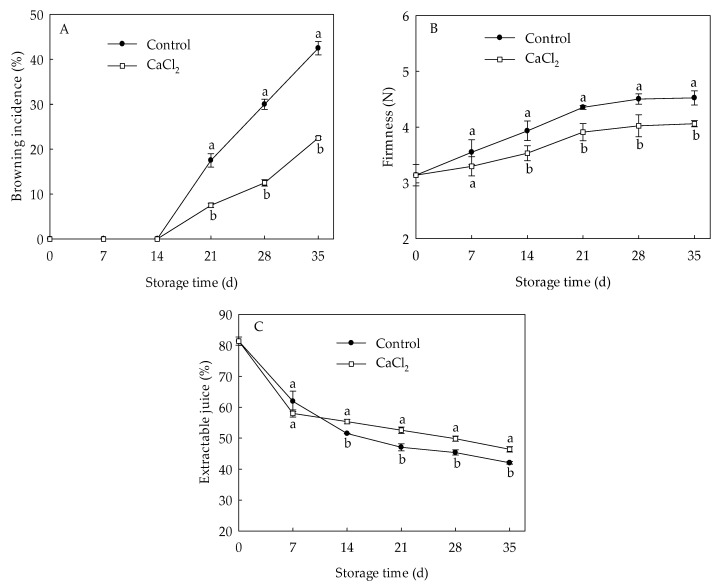
Browning index (**A**), firmness (**B**), and extractable juice (**C**) in loquat fruit stored at 1 °C after treatment with 1% CaCl_2_ or water (control). Vertical bars indicate the SE of mean (*n* = 3). The symbols a and b represent a significant difference in the two treatments (*p* < 0.05).

Internal browning in loquat fruit occurred on the 21st day of storage and increased sharply thereafter (Figure 1A). CaCl_2_ treatment significantly reduced the browning index under refrigeration. On day 35 of storage, browning index in CaCl_2_-treated fruit was 47.06% lower than that in control. The firmness in loquat fruit displayed an increase trend during the entire storage period (Figure 1B). CaCl_2_ treatment remarkably suppressed the upward trend of firmness compared to the control. Firmness in CaCl_2_-treated fruit was 10.18% lower compared with control on the 35th day of storage. Extractable juice of the control fruit declined gradually during cold storage (Figure 1C), whereas CaCl_2_-treated fruit displayed markedly higher extractable juice than that of the control from day 14 to day 35 of storage. These results indicated that CaCl_2_ treatment efficiently suppressed the increase in browning index, firmness, as well as retained higher extractable juice rate, consequently inhibiting CI symptoms of loquat fruit during cold storage.

### 3.2. Effects of CaCl_2_ Treatment on $O_{2}^{•-}$ Generation Rate and H_2_O_2_ Content of Loquat Fruit

**Figure 2:**
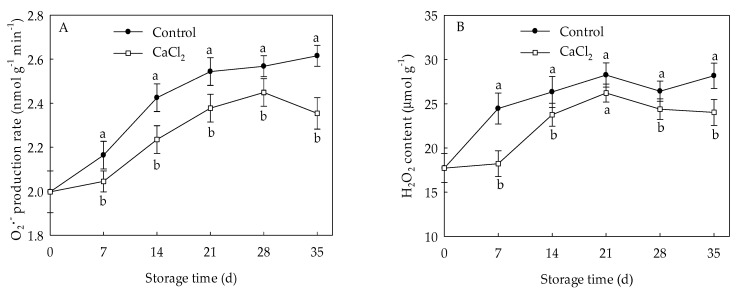
$O_{2}^{•-}$ generation rate (**A**) and H_2_O_2_ content (**B**) in loquat fruit stored at 1 °C after treatment with 1% CaCl_2_ or water (control). Vertical bars indicate the SE of mean (*n* = 3). The symbols a and b represent a significant difference in the two treatments (*p* < 0.05).

$O_{2}^{•-}$ generation rate and H_2_O_2_ level in control loquat fruit displayed overall increments during cold storage (Figure 2). However, compared to the control, the application of CaCl_2_ remarkably reduced the accumulation of $O_{2}^{•-}$ and H_2_O_2_ throughout the whole storage time. At the end of storage, the $O_{2}^{•-}$ generation rate and H_2_O_2_ content of CaCl_2_-treated fruit were 10.00% and 14.71% lower (*p* < 0.05) than those in control fruit, respectively.

### 3.3. Effects of CaCl_2_ Treatment on DPPH and $OH^{•}$ Radical Scavenging Capacity of Loquat Fruit

**Figure 3:**
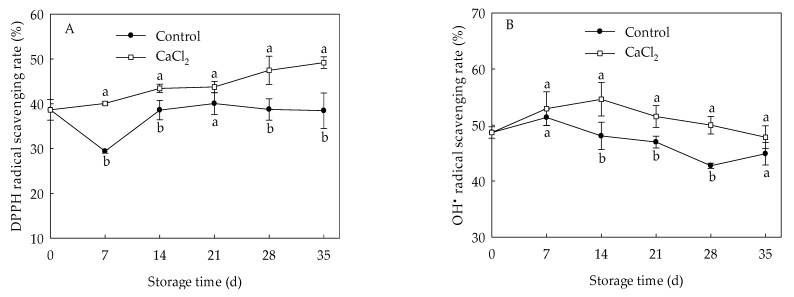
DPPH (**A**) and ${\mathrm{OH}}^{•}$ (**B**) radical scavenging capacity in loquat fruit stored at 1 °C after treatment with 1% CaCl_2_ or water (control). Vertical bars indicate the SE of mean (*n* = 3). The symbols a and b represent a significant difference in the two treatments (*p* < 0.05).

DPPH radical scavenging rate of control loquat fruit declined at the first 7 days of storage, and increased gradually from day 7 to day 21, then maintained relatively stable, while the CaCl_2_-treated fruit exhibited a gradually upward trend (Figure 3A). CaCl_2_ treatment maintained higher DPPH radical scavenging capacity within the entire storage period, and the DPPH radical scavenging rate was 27.85% higher in CaCl_2_-treated fruit than that of the control on the 35th day. The ${\mathrm{OH}}^{•}$ radical scavenging rate in control loquat fruit increased before day 7 of storage, but declined during day 7 to day 28, then displayed a slight increase (Figure 3B). However, the ${\mathrm{OH}}^{•}$ radical scavenging rate in the fruit treated with CaCl_2_ was higher compared to control fruit within the whole storage time, and presented a markedly (*p* < 0.05) higher ${\mathrm{OH}}^{•}$ radical scavenging rate during 14 d to 28 d of storage.

### 3.4. Effects of CaCl_2_ Treatment on CAT, SOD Activities and EjCAT, EjSOD Expressions of Loquat Fruit 

**Figure 4:**
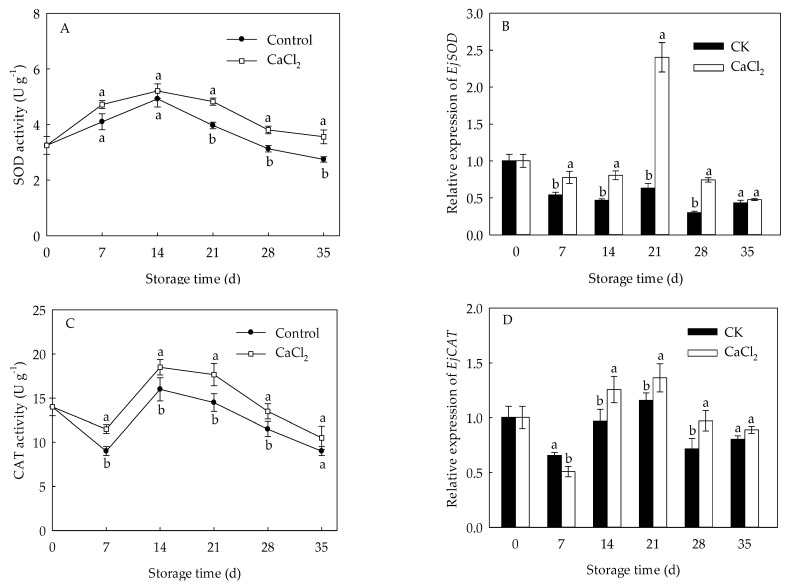
SOD activity (**A**), *EjSOD* expression (**B**), CAT activity (**C**) and *EjCAT* expression (**D**) in loquat fruit stored at 1 °C after treatment with 1% CaCl_2_ or water (control). Vertical bars indicate the SE of mean (*n* = 3). The symbols a and b represent a significant difference in the two treatments (*p* < 0.05).

The SOD activity of loquat fruit rose quickly from 0 to 14 d, but gradually decreased thereafter (Figure 4A). The CAT activity in loquat fruit dropped slowly from day 0 to day 7 of storage, but quickly rose from 7 d to 14 d, followed by a gradual declination (Figure 4C). CaCl_2_ treatment enhanced SOD and CAT activities throughout the whole storage period, with 21.61% and 21.84% higher than those in control on day 21, respectively. Expression levels of *EjSOD* and *EjCAT* presented a similar trend with that of SOD and CAT enzymes activities (Figure 4B,D). CaCl_2_ treatment up regulated the expression of *EjSOD* and *EjCAT*, which were approximate 2.80-fold and 17.83% higher on the 21st day of storage compared to control, respectively.

### 3.5. Effects of CaCl_2_ Treatment on Contents of AsA, GSH, GSSG and GSH/GSSG Ratio of Loquat Fruit

**Figure 5:**
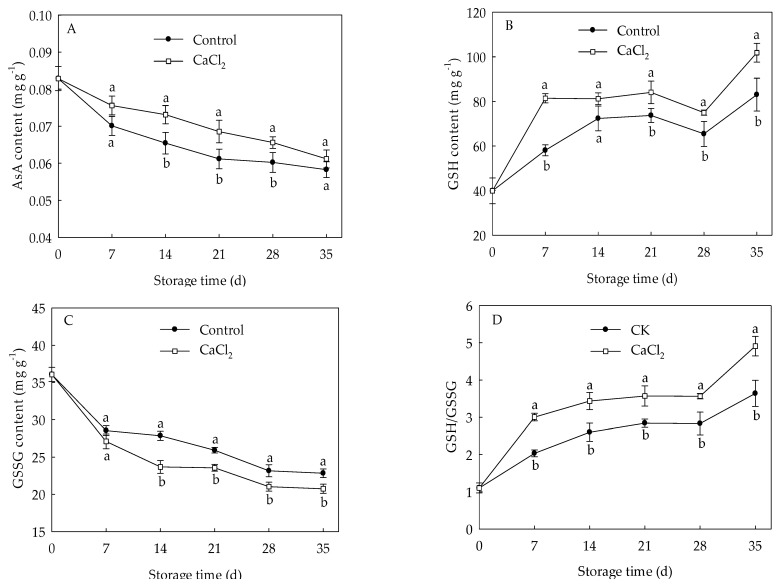
AsA (**A**), GSH (**B**), GSSG (**C**) contents and GSH/GSSG (**D**) in loquat fruit stored at 1 °C after treatment with 1% CaCl_2_ or water (control). Vertical bars indicate the SE of mean (*n* = 3). The symbols a and b represent a significant difference in the two treatments (*p* < 0.05).

The content of AsA in loquat fruit continuously declined with the extension of storage time (Figure 5A), whereas GSH content rose quickly from day 0 to day 21 of storage, then dropped slowly during 21–28 d, followed by a sharp increase (Figure 5B). Change in content of GSSG in loquat fruit was similar to that of AsA content (Figure 5C). On the contrary, GSH/GSSG in loquat fruit displayed a steady increase trend during cold storage (Figure 5D). CaCl_2_ treatment maintained higher AsA content and GSH content compared to the control, whereas GSSG content was lower in CaCl_2_-treated fruit than that in the control group. CaCl_2_ treatment remarkably increased GSH/GSSG within the entire storage time, and the GSH/GSSG in CaCl_2_-treated fruit was 34.92% higher on the 35th day of storage compared with control.

### 3.6. Effects of CaCl_2_ Treatment on Enzyme Activity and Gene Expression of APX, GR, DHAR, and MDHAR of Loquat Fruit

**Figure 6:**
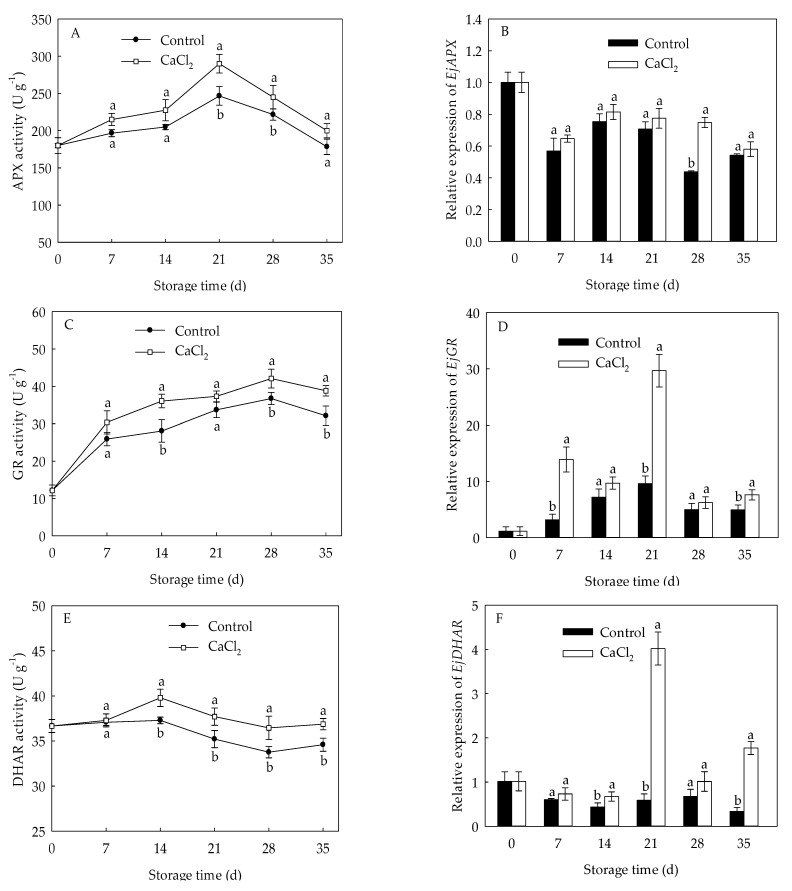

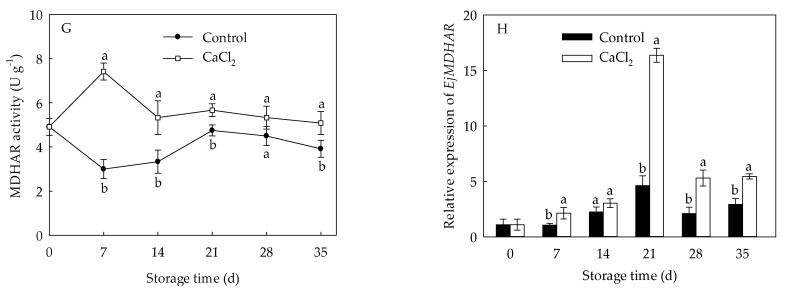
APX activity (**A**), *EjAPX* expression (**B**), GR activity (**C**), *EjGR* expression (**D**), DHAR activity (**E**), *EjDHAR* expression (**F**), MDHAR activity (**G**), and *EjMDHAR* expression (**H**) in loquat fruit stored at 1 °C after treatment with 1% CaCl_2_ or water (control). Vertical bars indicate the SE of mean (*n* = 3). The symbols a and b represent a significant difference in the two treatments (*p* < 0.05).

The APX activity in loquat fruit reached the peak on the 21st day of storage, and dropped quickly afterwards (Figure 6A). CaCl_2_-treated fruit exhibited higher APX activity compared to control fruit. Change of GR activity in loquat fruit displayed a similar trend with APX activity, but the maximum value reached on the 28th day (Figure 6C). Compared to control group, CaCl_2_ treatment maintained higher GR activity during cold storage, with a remarkable discrepancy from day 14 to day 35, except day 21 of storage.

The DHAR activity in control loquat fruit rose at first 14 d of storage, then declined during 14–28 d, followed by a slight increase (Figure 6E). However, CaCl_2_-treated fruit exhibited remarkably higher DHAR activity from day 14 to day 35 compared to the control fruit. MDHAR activity of control fruit decreased sharply initially, and rose during day 7 to day 21, then a slight drop during day 21 to day 35 (Figure 6G). CaCl_2_ treatment displayed remarkably higher MDHAR activity than that in control group during day 7 to day 35, except day 28.

The expression of *EjAPX* and *EjDHAR* in control loquat fruit generally showed a downward trend, but the CaCl_2_ treatment remained higher expression of *EjAPX* and *EjDHAR* throughout the entire storage period (Figure 6B,F). On day 21 of storage, *EjDHAR* expression in CaCl_2_-treated loquat fruit was about 6.82-fold of control. Expression of *EjGR* and *EjMDHAR* in loquat fruit increased and peaked at 21 d of storage, followed by an overall decline (Figure 6D,H). Compared with control fruit, CaCl_2_ treatment up regulated the expression of *EjGR* and *EjMDHAR* within the whole storage time. Expression of *EjGR* and *EjMDHAR* in the fruit treated with CaCl_2_ were 3.08-fold and 3.55-fold of those in control on the 21st day of storage, respectively.

## 4. Discussion

Chilling injury, manifested as internal browning, an unusual increase in firmness, and juiceless pulp, is a primary problem that significantly impacts the quality of loquat fruit during cold storage [10]. It is reported that Ca^2+^ acts as an important second messenger fulfilling a crucial role in protection against chilling injury in plants [13]. Previous studies have confirmed that exogenous CaCl_2_ treatment improved chilling tolerance in loquat fruit under low temperature storage [20]. In the present study, CaCl_2_ treatment efficiently suppressed the increase in browning index and firmness, and maintained higher extractable juice in loquat fruit (Figure 1), demonstrating CaCl_2_ exerts a positive influence in enhancing chilling tolerance in harvested loquat fruit. Similar result was also obtained in pear fruit [17] and pineapple fruit [25].

Destabilization of the cell membrane has been well-known as the primary reason of chilling injury in plants. The overproduction of ROS, including $O_{2}^{•-}$, H_2_O_2_ and hydroxyl radical, may result in the occurrence of CI [26,27]. SOD, CAT, and APX are considered to be vital ROS-eliminating enzymes which involve an alleviation of chilling injury [9]. SOD can convert the overproduced $O_{2}^{•-}$ into H_2_O_2_, while CAT and APX catalyze the H_2_O_2_ into H_2_O and O_2_ [28]. Antioxidant enzymes with higher activity and their integrated action have been demonstrated to be a part of the mechanism involved in the inhibition of oxidative damage and enhancement of cold tolerance in pear [29], blood orange [26], and banana [30]. In the current study, the accumulation of $O_{2}^{•-}$ and H_2_O_2_ in loquat fruit (Figure 2) demonstrated the membrane damage from accumulation of ROS under chilling stress, and CaCl_2_ treatment efficiently reduced ROS accumulation, leading to a lower membrane peroxidation in loquat fruit [20]. Compared with the control fruit, CaCl_2_-treated loquat fruit maintained higher DPPH and ${\mathrm{OH}}^{•}$ radical scavenging ability (Figure 3), which are two crucial parameters on the antioxidant capacity of plants [31]. Meanwhile, the SOD and CAT activities in loquat fruit rose during the early stage of storage, and CaCl_2_ treatment enhanced SOD and CAT activities within the whole storage period (Figure 4A,C). Moreover, the expression of *EjSOD* and *EjCAT* was also up regulated by CaCl_2_ treatment (Figure 4B,D). The increased activities and gene expressions of SOD and CAT might explain the lower levels of $O_{2}^{•-}$ and H_2_O_2_ in CaCl_2_-treated fruit. Zhang et al. [17] also reported that postharvest CaCl_2_ treatment promoted the activity and expression of SOD to against oxidative damage, inhibiting the browning in pear fruit after cold storage. Shi et al. [32] reported that CaCl_2_ treatment increased SOD and CAT activities to modulate redox homeostasis, contributing to improvement of chilling tolerance in bermudagrass. Similar results have also been confirmed in loquat fruit treated by methyl jasmonate [27]. These results indicated that the effect of CaCl_2_ on modulating the ROS homeostasis of loquat fruit was correlated to enhanced enzyme activities as well as gene expressions of SOD and CAT which, in turn, alleviated the occurrence of chilling injury.

The ASA–GSH cycle acts as a crucial antioxidant system to substantially scavenges H_2_O against oxidative damage, and APX, GR, DHAR, and MDHAR are key enzymes in this cycle [9]. AsA and GSH are products of the ASA-GSH cycle and important non-enzymatic antioxidants that are capable of directly or indirectly quenching ROS [7]. AsA is oxidized to the monodehydroascorbate (MDHA) radical, while APX converts H_2_O_2_ into the H_2_O with the help of AsA as an electron donor. MDHA regenerates AsA by MDHAR and is spontaneously converted to dehydroascorbate (DHA). DHA is reduced to AsA again through GSH, which leads to its oxidation to produce GSSG by DHAR. GSH regenerate from GSSG with catalyzation of GR [33]. Previous studies have demonstrated that enhanced AsA, GSH contents, and GSH/GSSG ratio are beneficial for the resistance to oxidative damage and improvement of chilling tolerance in plants [34,35]. Zhang et al. [19] confirmed that Ca^2+^ treatment could increase the activities of APX and GR to activate AsA-GSH cycle, and to effectively inhibit the increase in H_2_O_2_, thus, maintaining redox balance to better alleviate the occurrence of oxidative damage to green peppers under cold stress. In the current study, CaCl_2_-treated loquat fruit displayed higher levels of AsA and GSH contents (Figure 5A,B), lower GSSG content (Figure 5C), and retained higher GSH/GSSG ratio (Figure 5D) compared with the control fruit. Furthermore, CaCl_2_ enhanced the APX, GR, DHAR, and MDHAR activities in loquat fruit under cold storage (Figure 6A,C,E,G), which was conducive to maintaining a higher content of AsA, GSH and GSH/GSSG ratio. These findings were consistent with the result that ROS levels in CaCl_2_-treated fruit was lower than that in control (Figure 2). Similar results that AsA-GSH cycle played a crucial role in maintaining redox balance by loquat fruit under chilling injury were also reported by Cao et al. [10].

Additionally, the numerous reports showed that the increased expression of *APX*, *GR*, *DHAR*, *MDHAR* could alleviate oxidative injury due to their role in modulation of AsA–GSH cycle system in plants [33]. Song et al. [7] demonstrated that the mitigation of CI in peach fruit treated by hypobaric treatment was associated with its influence on activating the AsA-GSH cycle system through inducing *GR*, *MDHAR1*, and *APX* gene expressions to avoid oxidative damage. In the current study, CaCl_2_ treatment up regulated the *EjAPX*, *EjGR*, *EjDHAR*, and *EjMDHR* expressions in loquat fruit (Figure 6B,D,F,H), which exerted positive influence in the maintenance of high activities of antioxidant enzymes involved in AsA–GSH cycle, resulting in the inhibition of ROS accumulation and chilling injury symptoms. It was in agreement with previous work which stated that the up-regulated *CaAPX1*, *CaMDHAR1*, and *CaDHAR1* expressions in bell peppers by GSH treatment promoted activities of AsA-GSH cycle enzymes and regenerated AsA and GSH, consequently bringing about lower oxidative damage and alleviation of chilling injury [9]. Thus, we conclude that CaCl_2_ treatment could mitigate oxidative damage by activating AsA-GSH cycle system in loquat fruit during chilling injury.

## 5. Conclusions

In conclusion, CaCl_2_ treatment effectively enhanced cold tolerance and alleviated the occurrence of CI in loquat fruit. The inhibition in occurrence of CI by CaCl_2_ treatment might be ascribed to its capacity to reduce ROS accumulation by triggering of the antioxidant system including enzymatic and non-enzymatic antioxidant activities, thereby reducing cell membrane oxidative damage. These findings provide insight into the improvement of cold tolerance in loquat fruit through modulating ROS homeostasis by CaCl_2_ treatment. However, further molecular evidence is needed to support an explanation of how Ca^2+^ triggers low-temperature tolerance.

## Data Availability

Not applicable.

## Supplementary Materials

The following are available online at https://www.mdpi.com/article/10.3390/foods10071662/s1, Table S1. Primer sequences for Real-time PCR analysis.
