# Supplementary material for: Effects of CaCl2 Treatment Alleviates Chilling Injury of Loquat Fruit (Eribotrya japonica) by Modulating ROS Homeostasis
Source: Foods. 2021 Jul 19;10(7):1662. doi: 10.3390/foods10071662 (PMC8304281; doi:10.3390/foods10071662)
Supplement: Supplementary file 1 [file foods-10-01662-s001.zip › foods-1276301-SI.pdf]

Table S1. Primer sequences for Real-time PCR analysis

Gene	Forward Primer (5′ to 3′)	Reverse Primer (5′ to 3′)
EjSOD	ACTGTGATGCTTTCGTCCCC	CGGCGAAAGACATGGATTGG
EjCAT	GCTCCCAGTTAATGCTCCCA	GCACTTGTCACGCTTTCCAG
EjAPX	CGCCGAAACGAAAACAGGAG	TTGGCTTTCACTTCCTCGCA
EjGR	GTCGAAGTCATAGTGGGCCT	GCCTTTGCCCTCTGTACGAT
EjDHAR	CCTCCCAATGCCGTCGTAT	AACCATTCTGGCTTGTTGCC
EjMDHAR	ATAGGGCGATTGGTGCTTCC	TTACGGATCGGTTGTCGTGG
EjACT	AATGGAACTGGAATGGTCAAGGC	TGCCAGATCTTCTCCATGTCATCCCA
